# Synthesis and Application of Polyvinyl Alcohol (PVA) Micropowders for Antifouling Coatings

**DOI:** 10.3390/ma19071362

**Published:** 2026-03-30

**Authors:** Di Zhang, Dafu Wei, Xiang Xu, Yong Guan

**Affiliations:** Shanghai Key Laboratory of Advanced Polymeric Materials, School of Materials Science and Engineering, East China University of Science and Technology, Shanghai 200237, China; zhangdi1372@163.com (D.Z.); xiangxu@ecust.edu.cn (X.X.)

**Keywords:** hydrogel, antibacterial activity, coating, antifouling

## Abstract

An effective and facile emulsion polymerization method was developed for the preparation of highly antibacterial hydrogel. In this study, polyhexamethylene guanidine (PHMG) was successfully cross-linked with polyvinyl alcohol (PVA) to form hydrogel micropowders with exceptional antibacterial properties. Subsequently, to improve the mechanical properties of the hydrogel, the hydrogel micropowders cross-linked with the antibacterial agent were combined with epoxy resin and coated onto stainless steel plates to form composite coatings. FT-IR spectroscopy was used to analyze the composition of the samples, confirming the successful reaction between PVA and PHMG. Bacterial inhibition assays demonstrated that the product exhibited an inhibition rate exceeding 99.99% against both *Escherichia coli* (*E. coli*) and *Staphylococcus aureus* (*S. aureus*) when the PHMG content exceeded 2.0 wt%. The anti-protein adsorption and algae resistance tests confirm the good antifouling performance of the coatings. These results highlight the potential of this material for marine antifouling applications.

## 1. Introduction

The scientific evolution of hydrogels traces back to the last century, with their initial applications driven by fundamental human needs for soft and hydrophilic materials. From early uses in soft contact lenses [1] and hygiene products [2] to contemporary applications in controlled drug delivery and tissue engineering scaffolds [3], hydrogels have attracted considerable attention owing to their high water content microenvironment, a characteristic that closely mimics the soft tissues of biological systems. Hydrogels can be tailored to exhibit a wide range of physicochemical properties by modifying their network structures, enabling customization to meet diverse application requirements [4,5,6]. As a result, hydrogels have been widely applied across various scientific and industrial fields [7,8,9]. Among numerous polymeric materials, polyvinyl alcohol (PVA) is widely regarded as a prominent candidate for hydrogel applications, owing to its exceptional biocompatibility, tunable cross-linked networks with remarkable mechanical toughness, and high density of modifiable hydroxyl functional groups.

PVA-based hydrogels are recognized as robust drug delivery matrices due to their non-toxicity, biocompatibility, and distinctive gel-like properties, enabling the efficient encapsulation and controlled release of diverse therapeutics to improve efficacy and reduce side effects [10,11]. Fabián Martínez-Gómez et al. fabricated sodium alginate–polyvinyl alcohol (SA-PVA) hydrogels via a simple and low-cost freeze–thaw cycling approach. UV-Vis spectroscopy was employed to evaluate the encapsulation efficiency and release kinetics of metformin hydrochloride. The results demonstrated that the SA-PVA blend hydrogels possess pH-responsive properties, which enable effective protection of the encapsulated drug under acidic conditions simulating the gastric environment [12].

In the field of tissue engineering, hydrogels have demonstrated considerable potential for the repair and regeneration of diverse tissues and organs, positioning them as a highly promising area of frontier research. Hydrogels offer a favorable microenvironment that supports cell adhesion, proliferation, and differentiation, rendering them ideal scaffold materials for diverse tissue engineering applications such as cartilage, bone, and vascular tissue regeneration. Moreover, the smooth and compliant nature of PVA hydrogels closely mimics the mechanical properties of soft biological tissues, endowing them with particular advantages in applications including artificial joints and contact lenses [13,14].

By incorporating conductive components, PVA-based hydrogels can be engineered into conductive hydrogels, thereby extending their potential for applications in supercapacitors and strain sensors. These functionalized hydrogels retain the desirable characteristics of conventional hydrogels—including flexibility, biocompatibility, and high water content—while gaining tunable electrical conductivity. This unique combination of properties makes PVA-based conductive hydrogels highly promising for integration into advanced flexible electronic devices and intelligent technological systems [15].

Nevertheless, the applications of hydrogels extend well beyond these domains. In recent years, hydrogels have emerged as promising materials in the highly challenging field of marine antifouling, where they demonstrate unique advantages and practical value. The colonization of marine organisms on submerged surfaces leads to detrimental consequences such as: (1) reduced vessel hydrodynamic efficiency, (2) increased fuel consumption, and (3) accelerated structural corrosion. These impacts collectively contribute to substantial economic losses and environmental burdens [16,17,18,19]. Consequently, developing effective antifouling strategies to mitigate marine organism colonization has emerged as a critical research priority in marine science and engineering. Hydrogel coatings show great potential as environmentally friendly material for marine antifouling [20]. The porous structure of hydrogels enables water absorption, forming a stable hydrated interfacial layer. This layer functions as both a physical barrier and an energy barrier, effectively preventing protein adsorption [21], fouling deposition [22] and microbial attachment [23]. Furthermore, most marine organisms exhibit preferential adhesion to rigid surfaces. Given their low elastic modulus, hydrogels inherently resist biofouling by minimizing surface attachment [24]. Among emerging marine antifouling coating materials, hydrogels are regarded as one of the most promising candidates.

Nevertheless, the practical implementation of hydrogel materials in marine antifouling engineering encounters substantial challenges. The key bottlenecks comprise the deterioration of mechanical strength caused by water absorption and swelling, as well as inadequate interfacial adhesion toward substrates. These drawbacks severely restrict the large-scale application of such materials in marine antifouling systems. Therefore, the development of effective strategies to reinforce hydrogel coatings is urgently required. By rationally optimizing the interfacial bonding mechanism between the coating and substrate, the mechanical properties and adhesive stability of hydrogels can be systematically enhanced [25].

Hydrogels are conventionally produced in bulk forms, which may not satisfy the practical demands of certain specialized applications. To overcome this limitation, various strategies have been developed for fabricating hydrogel micropowders. These micropowders are composed of micrometer-sized hydrogel particles, typically with diameters ranging from 1 to 1000 μm. Furthermore, the application potential of hydrogel micropowders can be enhanced through subsequent chemical modifications [26]. Several techniques are available for synthesizing hydrogel micropowders, including ionic gelation [27], extrusion [28,29], spray drying [30], and emulsification [31,32]. Among these, emulsification is the most widely adopted method owing to its reproducibility and operational simplicity. An emulsion consists of two immiscible liquids, in which one phase is dispersed as droplets within the other, forming a thermodynamically unstable system. To enhance stability, emulsifiers or surfactants are typically introduced to lower the interfacial free energy, thus conferring kinetic stability. Due to their simple operation, low cost, and high reproducibility, emulsion-based approaches are widely utilized for the fabrication of microspheres [33]. Emulsions can be classified into various types—including ethanol-in-oil, water-in-oil, or oil-in-water—according to the nature of the dispersed and continuous phases. The choice of a suitable emulsion system relies on the specific materials employed and the target application.

The main objective of this study is to develop a simple and efficient method for the emulsion polymerization of polyhexamethylene guanidine (PHMG) and PVA, by which antibacterial PVA hydrogel micropowders were successfully prepared and then incorporated with epoxy resin (EP) coatings for antifouling applications. The as-prepared material exhibited antibacterial properties as well as resistance to protein adsorption and algal adhesion. When introduced into EP coatings, PHMG, as a highly effective antibacterial agent, showed outstanding bactericidal activity against both *Escherichia coli* (*E. coli*) and *Staphylococcus aureus* (*S. aureus*), with inhibition rates exceeding 99.99%. Notably, the amino groups of PHMG participated in the curing reaction of epoxy resin, which significantly enhanced the interfacial adhesion between the hydrogel micropowders and the epoxy matrix. This approach fully releases the antibacterial activity of PHMG while simultaneously improving the durability, stability, and overall mechanical properties of the coatings.

## 2. Experimental Section

### 2.1. Materials

All chemicals and solvents were used as received without further purification. PVA (Type 1799) was obtained from Shanghai Titan Technology Co., Ltd., Shanghai, China. Bovine serum albumin (BSA), Span-80, Tween-80, and glutaraldehyde (50% aqueous solution) were purchased from Shanghai Aladdin Biochemical Technology Co., Ltd., Shanghai, China. PHMG was synthesized according to the reported method. SiO_2_ (1 μm diameter) was supplied by Sinopharm Chemical Reagent Co., Ltd., Shanghai, China. Microorganisms including *E. coli* (ATCC 8099), *S. aureus* (ATCC 6538), and *Chlorella vulgaris* were acquired from the Shanghai Center for Disease Control and Prevention (Shanghai, China) and Nanjing Haierse Biotechnology Co., Ltd. (Nanjing, China), respectively.

### 2.2. Preparation of 10% PVA and 20% PHMG Aqueous Solutions

A 500 mL three-necked flask equipped with a mechanical stirrer was charged with 360 g of deionized water and placed into a thermostatted oil bath. Under constant stirring at 300 rpm, 40 g of PVA pellets was added gradually at room temperature (25 °C) and stirred for 30 min to facilitate dissolution. Subsequently, the system was heated to 95 °C and stirred continuously for 6 h until a uniform, transparent solution was obtained.

80 g of deionized water and 20 g of PHMG were poured into a 200 mL beaker and stirred magnetically at room temperature until PHMG was completely dissolved.

### 2.3. Synthesis of PVA-PHMG-X

PVA-PHMG hydrogel micropowders with different amounts of PHMG were prepared. The recipes are summarized in Table 1.

**Figure 1:**
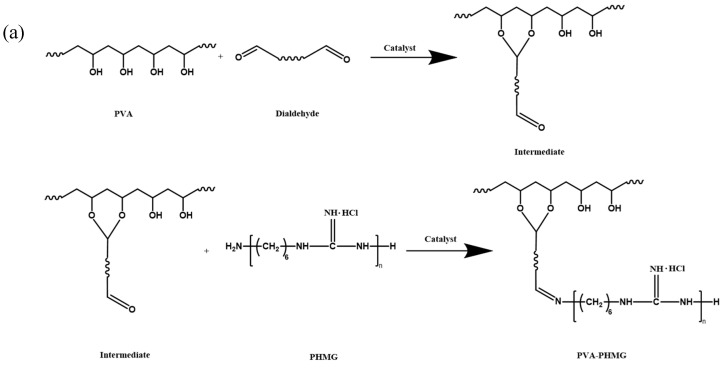

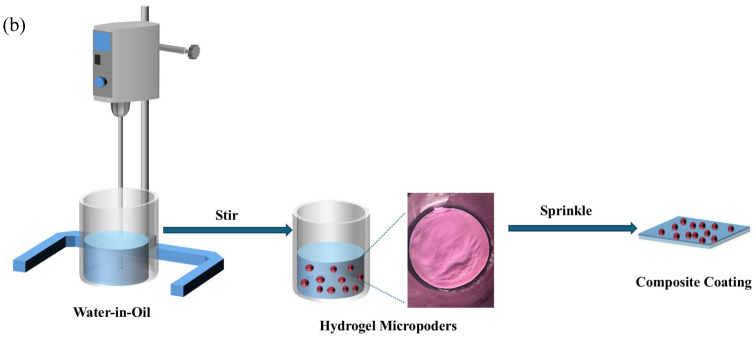
(**a**) Reaction mechanism for the synthesis of antibacterial hydrogels via water-in-oil (W/O) emulsion polymerization. (**b**) Reaction device diagram of PVA-PHMG hydrogel micropowders.

The hydrogel micropowders were prepared through a water-in-oil emulsion polymerization method. Firstly, a stable oil phase was prepared by dissolving 2.4 g of Span 80 and 1.2 g of Tween 80 in 400 mL of dimethicone oil under continuous stirring for 30 min. Simultaneously, an aqueous phase was formulated by mixing 20 g PVA solution with 2 g of PHMG aqueous solution and 78 g of deionized water, followed by the addition of 2 g of glutaraldehyde (GA) as crosslinker. The aqueous phase was then slowly added into the oil phase system to form a stable water-in-oil emulsion. The pH of the system was sequentially adjusted, first to acidic using HCl for acid-catalyzed crosslinking (2 h), and then secondly to slightly alkaline using NaOH for base-catalyzed curing (2 h). Following the reaction, the crude product was filtered through a 200-mesh stainless steel sieve to collect the hydrogel micropowders. Sequential washing was then conducted as follows: (1) hexane was used to remove residual dimethicone oil, followed by (2) deionized water to eliminate surfactants and unreacted PHMG. The purified hydrogel micropowders were subsequently vacuum-dried at 40 °C for 24 h, yielding the final PVA-PHMG hydrogel micropowders. A schematic diagram of the experimental setup and synthetic procedure is depicted in Figure 1.

Figure 1a schematically illustrates the crosslinking mechanism among PVA, PHMG, and GA. While the diagram shows a simplified reaction pathway involving a single GA molecule, the actual crosslinking process is significantly more complex. As a bifunctional crosslinking agent, GA can simultaneously: (1) bridge PVA and PHMG chains via Schiff base formation and acetal linkages, and (2) induce intramolecular crosslinking within individual PVA or PHMG networks. These multivalent interactions ultimately give rise to a three-dimensional, mesh-like architecture with improved structural stability and integrity.

### 2.4. Synthesis of EP/PVA-PHMG-X

The EP and curing agent were initially homogenized via mechanical stirring, followed by ultrasonic degassing to remove entrapped air bubbles. The resulting bubble-free resin mixture was uniformly coated onto stainless steel substrates using a precision film applicator, with a controlled thickness of 400 ± 20 μm. The as-synthesized PVA-PHMG-X hydrogel micropowders were then evenly dispersed onto the coated surface. Finally, the coated substrates were placed in a vacuum drying oven and thermally cured under a programmed heating schedule (holding for 2 h) to obtain the final epoxy–hydrogel composite coatings.

### 2.5. Characterization of Materials

#### 2.5.1. Fourier Transform Infrared Spectroscopy (FTIR)

Fourier transform infrared spectroscopy (FTIR, Nicolet 5700, Thermo Fisher Scientific, Waltham, MA, USA) is used to determine the composition of the cross-linked micropowders. The infrared spectra of micropowders were scanned in the range of 500–4000 cm^−1^.

#### 2.5.2. Ultraviolet Absorption Spectrum Test

The leaching rate of PHMG was determined by UV–vis absorption spectroscopy. Calibration was performed using a series of aqueous PHMG solutions with varying concentrations, and their corresponding UV absorption spectra were recorded. A characteristic absorption peak was observed at 192 nm. A linear calibration curve of PHMG absorbance versus concentration was then established via linear regression, as presented below.

Abs_192nm_ = 0.11439 + 0.04035C_PHMG_

The detailed calibration procedure for the UV–vis standard curve is provided in the Section S1 of the Supporting Information.

A total of 0.5 g of the PVA-PHMG sample was weighed and put into 100 mL of deionized water for 24 h, followed by magnetic stirring for 24 h. The resulting aqueous solution was collected for subsequent measurement. The concentration of PHMG in the samples after immersion was calculated by the relationship curve. The leaching rate of the sample was calculated according to the following formula.

$$\mathrm{Leaching}\;\mathrm{rate}=\frac{C_{PHMG}V_{W}}{W}\times 100\%$$

C_PHMG_ denotes the concentration of PHMG in the soaking solution, V_W_ denotes the volume of the soaking solution, and W denotes the mass of PHMG added.

#### 2.5.3. SEM and EDS Characterization Methods

Scanning electron microscopy (SEM, Hitachi S4800N, Tokyo, Japan) operated at an acceleration voltage of 15 kV and was used to observe the surface morphology of the hydrogel micropowders, as well as the surface and cross-sectional morphologies of the composite coatings. Additionally, energy dispersive spectroscopy (EDS) was employed to investigate the distribution characteristics of chlorine (Cl) on the surface of the polyvinyl alcohol composite coatings (EP/PVA-PMG-X).

#### 2.5.4. Thermal Performance Test

Thermal stability of the samples was evaluated by thermogravimetric analysis (TGA) (STA409PC, NETZSCH, Waldkraiburg, Germany). Approximately 5–10 mg of dried sample was weighed and placed in an alumina crucible. The analysis was performed under a nitrogen flow of 20 mL/min, with the sample heated from 30 °C to 600 °C at a heating rate of 10 °C·min^−1^. The mass loss as a function of temperature was recorded to characterize the thermal stability of the samples.

#### 2.5.5. Water Absorbency Test

The water absorption of the coatings was determined via the gravimetric method. Briefly, the coatings were immersed in deionized water and withdrawn after a predetermined time interval. Surface-adhered water was gently blotted with filter paper, and the sample was immediately weighed. The water absorption of the coatings were calculated using the equation presented below.

$$Water\;absorption=\frac{m_{2}-m_{1}}{m}\times 100\%$$

m_1_ and m_2_ are the masses of the coatings before and after water absorption, respectively, and m is the mass of hydrogel micropowders added to the coatings, which is 0.1 g.

#### 2.5.6. Antibacterial Test

The antibacterial activities of the micropowders and coatings were evaluated using the shaking flask method and the film application method, respectively. For the shaking flask method, the samples and bacterial suspensions were mixed and co-incubated under constant-temperature shaking to ensure sufficient dynamic contact between the bacteria and samples. After incubation, the bacterial suspension was serially diluted by factors of 10, 100, and 1000. A 0.1 mL aliquot of each diluted suspension was inoculated onto LB agar plates and spread evenly. The plates were then incubated upside down at 37 °C for 24 h in a constant-temperature incubator. Following incubation, the number of viable bacteria was determined by the plate counting method, and the antibacterial or bactericidal efficiency was calculated accordingly. For the film application method, bacterial suspensions were sandwiched between the sample surface and a sterile film for static contact cultivation. After incubation at a constant temperature, the samples and cover films were rinsed repeatedly with 1 mL of PBS, and all eluates were collected. Then the same procedure was employed as described for the shaking flask method. Bacterial colony growth was observed to evaluate the antibacterial activity of the material surface. The leaching behavior of PHMG from the coatings was assessed via the inhibition zone method. *E. coli* and *S. aureus* were chosen as model bacterial strains. All glassware was autoclaved for sterilization prior to the antibacterial assays.

#### 2.5.7. Anti-Protein Adsorption Test

During the initial stage of conditioning film formation, biofouling typically commences with the adsorption of polysaccharides and proteins onto material surfaces, leading to the development of a primary conditioning layer. This process leads to an increase in surface roughness, thereby promoting further microbial colonization and accelerating biofouling development [34]. Accordingly, suppressing protein adsorption is critical to impeding subsequent biological contamination. Extensive studies have demonstrated that the protein resistance of materials is closely correlated with their surface energy and wettability. Benefiting from their intrinsic hydrophilic character, hydrogels can significantly reduce protein adhesion, thus endowing them with excellent antifouling performance toward protein adsorption.

Hydrogel coating samples (5 cm × 5 cm) in the fully swollen state were immersed in 15 mL of phosphate-buffered saline (PBS) containing bovine serum albumin (BSA) at a concentration of 0.5 g L^−1^. After incubation for 24 h, the samples were removed, and the absorbance of the residual BSA solution at 278 nm was determined by UV–vis spectroscopy. The amount of adsorbed BSA was then calculated using the BSA calibration curve.

#### 2.5.8. Algae Resistance Test

During marine biofouling development, the adhesion of microorganisms and protein typically precedes colonization by algae, which subsequently become a dominant component of fouling communities. In this work, *Chlorella*—a widely distributed algal species—was employed as a model organism to assess the antifouling performance of hydrogel coatings against algal adhesion. To simulate natural marine conditions and support normal algal growth and metabolism, *Chlorella* algae strains were added to artificial seawater and cultured at 25 °C for 7 days; all experiments were performed at room temperature under a 12 h light/12 h dark photoperiod. Hydrogel-coated substrates were immersed in *Chlorella* suspensions for 14 days. Following incubation, to simulate the marine scouring environment, the samples were gently rinsed with artificial seawater to remove weakly adhered algae, and the degree of algal adhesion on the coating surfaces was subsequently characterized.

## 3. Results and Discussion

### 3.1. Synthesis of Samples

PVA hydrogel micropowders were synthesized via a cross-linking reaction, in which PHMG, serving as an antibacterial agent, was covalently bound to the PVA molecular chains. Following the reaction, the as-prepared hydrogel micropowders were thoroughly washed to eliminate unreacted PHMG and residual impurities, and subsequently vacuum-dried to remove entrapped moisture.

**Figure 2:**
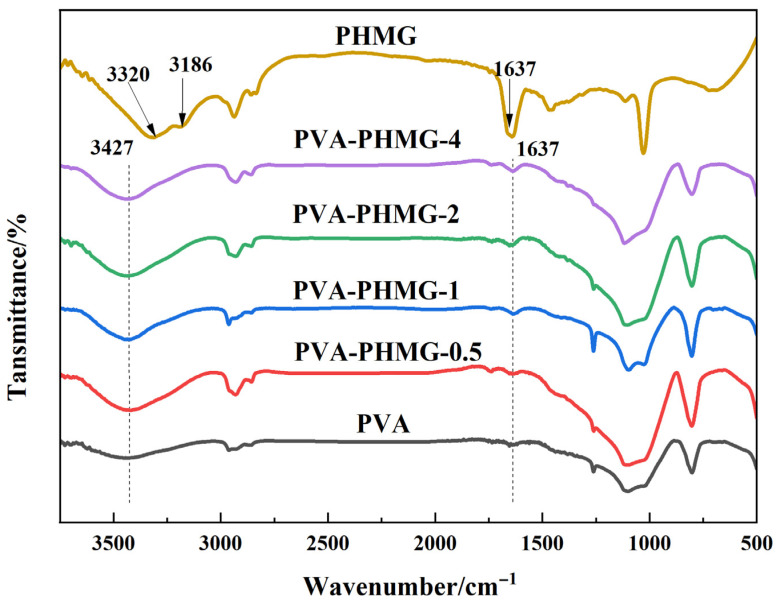
FTIR spectra of PVA, PHMG and PVA-PHMG-X.

The FTIR spectra of PVA, PHMG, and the functionalized PVA-PHMG micropowders are displayed in Figure 2. The spectrum of the neat PVA hydrogel shows a broad and intense absorption band at 3000–3600 cm^−1^, which is ascribed to O–H stretching vibrations [35], reflecting the extensive hydrogen-bonding network within the hydrogel matrix. For PHMG, the absorption bands at 3320 cm^−1^ and 3186 cm^−1^ are attributed to N–H stretching vibrations of the amine groups (–NH_2_), whereas the peak at 1637 cm^−1^ is assigned to the C=N stretching vibration of the guanidine moiety [36].

In the FTIR spectrum of PVA-PHMG-X, a characteristic absorption band emerges at 3427 cm^−1^, arising from the overlapping stretching vibrations of hydroxyl groups (–OH) in PVA and amine groups (–NH_2_) in PHMG. Furthermore, the characteristic peak at 1637 cm^−1^ verifies the presence of the guanidine moiety, confirming the successful immobilization of PHMG onto the PVA backbone.

The activation energy and reaction mechanism were simulated using Gaussian 16 W software (Rev: C.01) with the corresponding results depicted in the Section S2 of the Supporting Information.

### 3.2. Ultraviolet Absorption Spectrum

The leaching rate was quantitatively determined as summarized in Table 2. The results reveal that trace levels of residual PHMG after washing, indicating the low leachability of the immobilized PHMG. This demonstrates that the antibacterial activity of the hydrogel micropowders originate not from loosely adsorbed or unbound PHMG on the surface but from the fraction covalently bonded onto the PVA matrix. Accordingly, the chemically bonded PHMG is believed to play a dominant role in the antibacterial mechanism of the as-prepared micropowders.

### 3.3. SEM Morphologies and EDS Analysis

**Figure 3:**
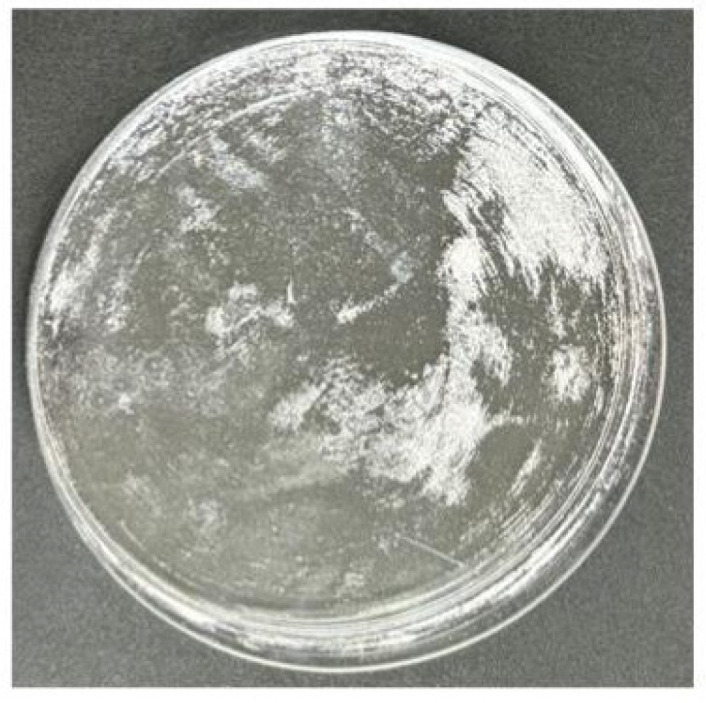
Macroscopic morphology of the micropowders.

**Figure 4:**
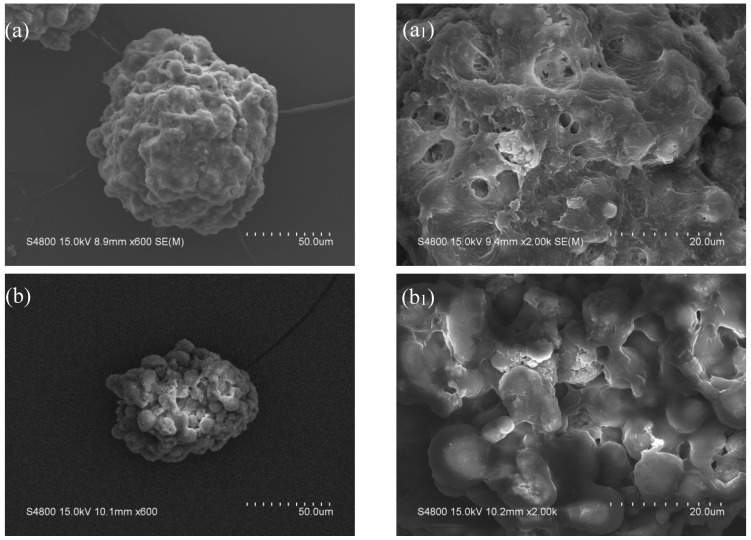

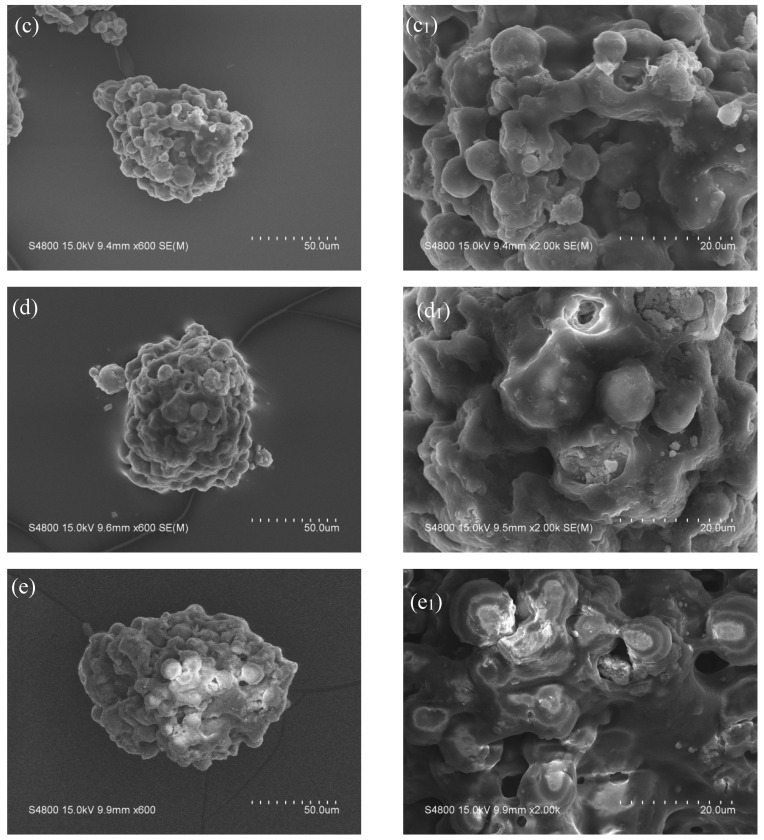
SEM images of a single dry PVA-PHMG hydrogel micropowder. (**a**,**a_1_**) PVA; (**b**,**b_1_**) PVA-PHMG-0.5; (**c**,**c_1_**) PVA-PHMG-1; (**d**,**d_1_**) PVA-PHMG-2; (**e**,**e_1_**) PVA-PHMG-4.

The macroscopic appearance and microscopic surface morphologies of the micropowders are displayed in Figure 3 and Figure 4, respectively. The neat PVA hydrogel micropowders (without antibacterial agent) display a nearly spherical morphology, characterized by well-defined and uniformly distributed porous structures with interconnected pore channels. This highly ordered porous network represents a typical feature of neat PVA hydrogels, which is conducive to water absorption. In contrast, the antibacterial agent-loaded hydrogel micropowders exhibit an irregular shape with a rough and uneven surface. The pore structures become indistinct and shallower, which can be ascribed to the deposition and distribution of the antibacterial agent within the hydrogel matrix. Some residual porous features are still observable, but the overall porosity is reduced.

Furthermore, this irregular and rough morphology is expected to provide a larger specific surface area, thereby enhancing the interfacial adhesion between the micropowders and the resin matrix, which is beneficial for functional performance of the resulting composite materials.

**Figure 5:**
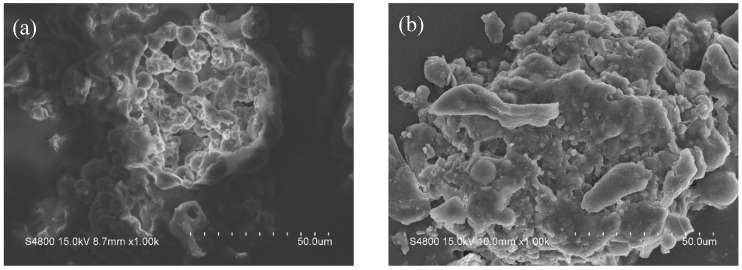

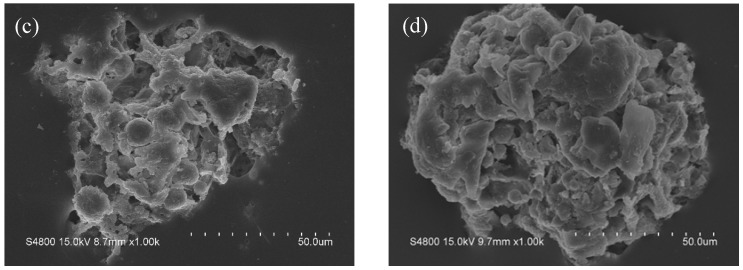
SEM surface morphology of hydrogel micropowder–epoxy resin composite coatings. (**a**) EP/PVA-PHMG-0.5; (**b**) EP/PVA-PHMG-1; (**c**) EP/PVA-PHMG-2; (**d**) EP/PVA-PHMG-4.

**Figure 6:**
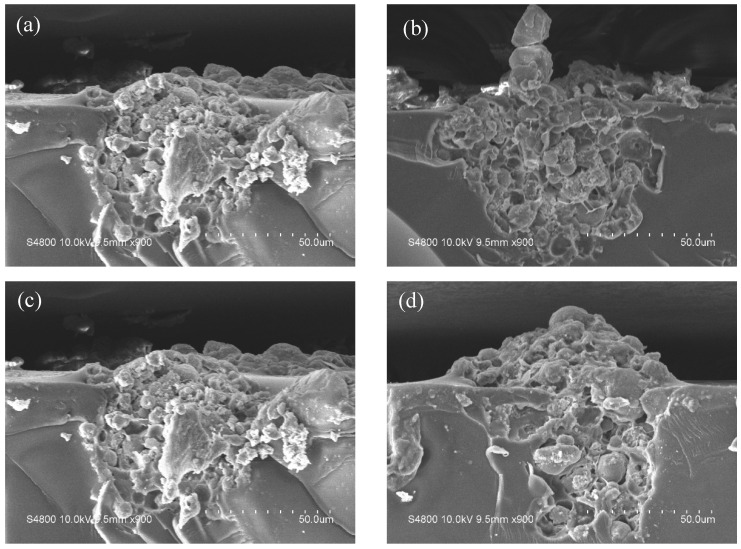
SEM cross-section morphology of hydrogel micropowder–epoxy resin composite coatings. (**a**) EP/PVA-PHMG-0.5; (**b**) EP/PVA-PHMG-1; (**c**) EP/PVA-PHMG-2; (**d**) EP/PVA-PHMG-4.

SEM images of the composite coatings are depicted in Figure 5 and Figure 6. The graphs demonstrate that the hydrogel particles are partially embedded within the coating matrix and partially exposed at the outermost surface. This unique structure guarantees that the hydrogel micropowder is stably anchored within the coatings.

**Figure 7:**
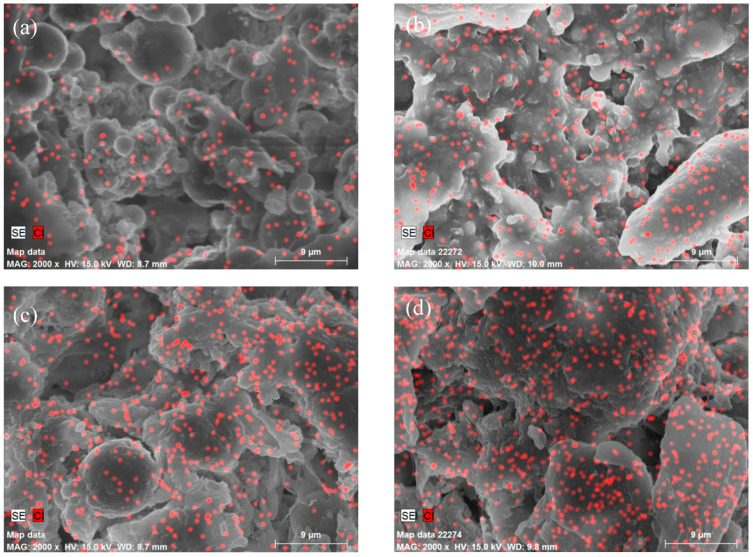
EDS spectra of hydrogel micropowder–epoxy resin composite coatings. (**a**) EP/PVA-PHMG-0.5; (**b**) EP/PVA-PHMG-1; (**c**) EP/PVA-PHMG-2; (**d**) EP/PVA-PHMG-4.

Figure 7 shows the EDS elemental mapping images of the composite coatings. Chlorine (Cl) elements were uniformly distributed on the coating surface, and the intensity of the Cl signal gradually increased with the increasing PHMG dosage during preparation. These results verify the successful introduction and homogeneous dispersion of PHMG in the coating matrix. Moreover, the positive correlation between PHMG content and Cl signal intensity confirms that increasing amounts of PHMG were stably combined with the PVA matrix, thus endowing the coating with effective antibacterial activity.

### 3.4. Thermal Performance Analysis

**Figure 8:**
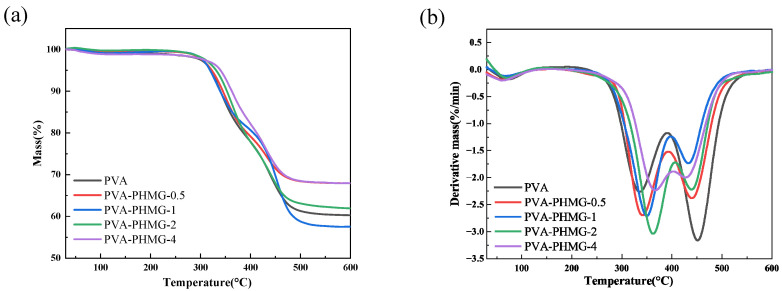
TGA spectra of PVA and PVA-PHMG-X: (**a**) TG and (**b**) DTG.

Thermal degradation behaviors of the samples were investigated via TGA, as displayed in Figure 8, with the corresponding thermal decomposition data summarized in Table 3. As listed in Table 3, the initial decomposition temperature, defined as the temperature at 5% weight loss (Td_5_), increases with increasing PHMG content in the micropowders. This observation indicates that the incorporation of higher contents of PHMG and glutaraldehyde enhances the cross-linking density of the hydrogel network, thus improving the thermal stability of the material.

### 3.5. Water Absorption Analysis

**Figure 9:**
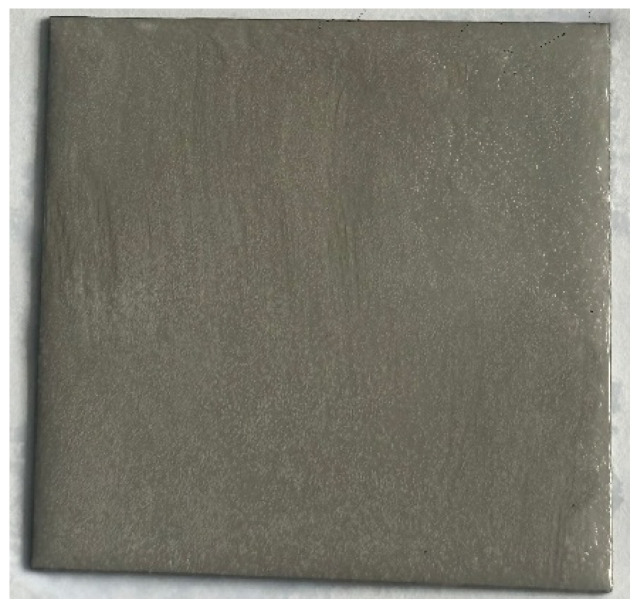
Picture of EP/PVA-PHMG-2 hydrogel coating after water absorption.

The water absorption of the coatings composed of 0.1 g hydrogel micropowders and 1 g epoxy resin was calculated (Table 4). The results indicate that the EP/PVA-PHMG-0.5 coating exhibits the highest water absorption capacity, while the EP/PVA-PHMG-4 coating shows the lowest, a trend consistent with the thermal analysis findings. The hydrated state of the EP/PVA-PHMG-2 coating after water absorption is illustrated in Figure 9. Furthermore, morphological observation of the micropowders after immersion in water confirmed their stability and insolubility, further supporting the presence of a cross-linked network structure within the hydrogel micropowders.

### 3.6. Antibacterial Activity

PHMG exhibits strong antibacterial activity. The molecular structure of PHMG possesses abundant cationic guanidino groups. These cationic moieties can interact with negatively charged teichoic acids or lipopolysaccharides via electrostatic attraction, thereby disrupting the cell membrane and exerting a bactericidal effect [37].

**Figure 10:**
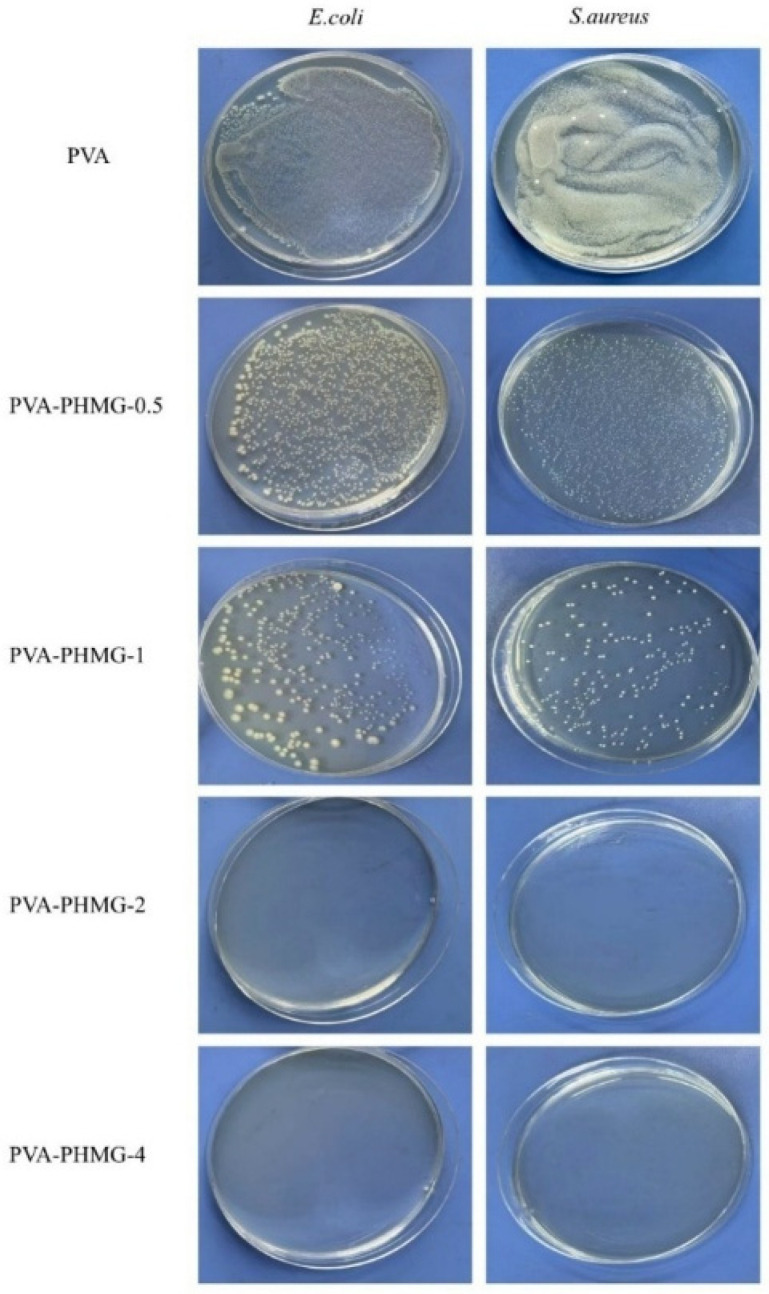
Antibacterial pictures of PVA-PHMG-X against *E. coli* and *S. aureus* tested by the shaking flask method.

**Figure 11:**
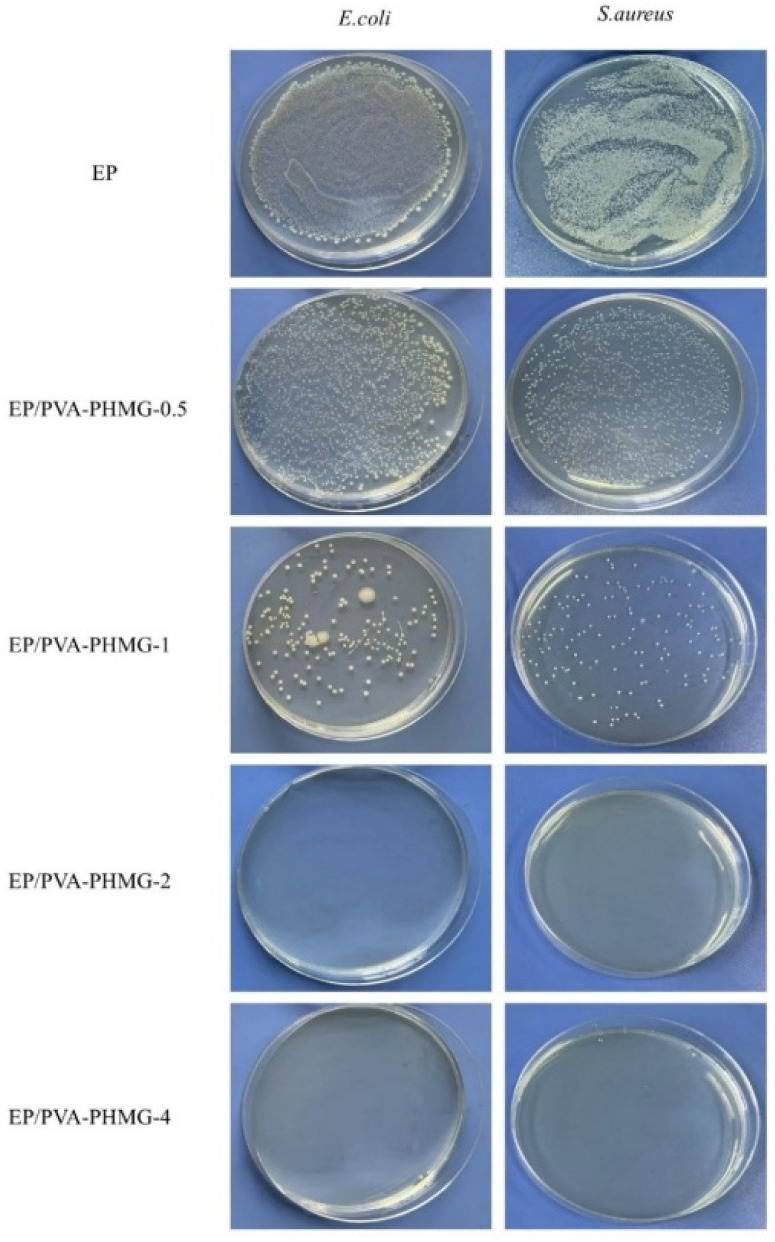
Antibacterial pictures of EP/PVA-PHMG-X against *E. coli* and *S. aureus* tested by the film application method.

**Figure 12:**
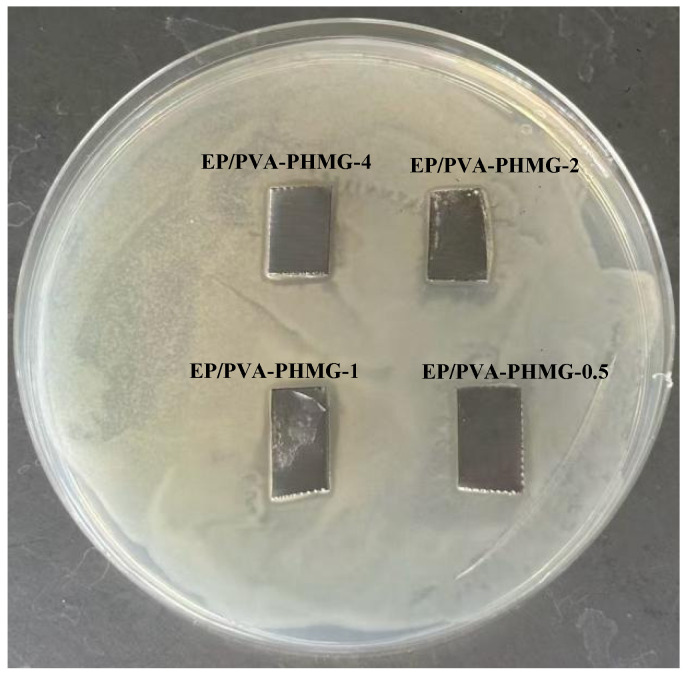
Antibacterial picture of EP/PVA-PHMG-X tested by the inhibition zone method against *E. coli*.

The antibacterial properties of the synthesized hydrogel micropowders were evaluated using the shaking flask method (Table 5), while those of the EP/PVA-PHMG-X coatings were assessed via the film application method (Table 6). The release behavior of PHMG was examined using the inhibition zone method. The corresponding results are presented in Figure 10, Figure 11 and Figure 12.

It can be concluded that the micropowders and coatings display remarkable antibacterial activity against both *E. coli* and *S. aureus* when the amount of added PHMG solution reaches 2 wt%. All coated samples containing PHMG generate inhibition zones, albeit with relatively small diameters, suggesting the presence of only a limited quantity of free PHMG within the coating matrix.

Owing to the high water solubility of PHMG, residual PHMG located on the surface of the hydrogel micropowders can gradually diffuse into the agar medium. During diffusion, the released PHMG interacts with *E. coli* and exerts efficient antibacterial effects by destroying bacterial cellular structures or interfering with metabolic processes, ultimately leading to the formation of an inhibition zone around the sample. These results are consistent with the UV spectroscopic analysis, further verifying that only a little of the PHMG remains adsorbed on the surfaces of the micropowders.

### 3.7. Anti-Protein Adsorption Analysis

The determination of the BSA standard curve is documented in the Section S3 of the Supporting Information.

As listed in Table 7, the absorbance values of the samples at 278 nm differed noticeably. The EP/PVA-PHMG-4 sample exhibited the highest absorbance (0.3187), whereas the neat EP sample showed the lowest value (0.2658). With increasing PHMG loading, the concentration of residual BSA in the solution increased, indicating improved resistance to BSA adsorption. These results demonstrate that PHMG effectively suppresses protein adsorption onto the surface of the coatings, thus enhancing its antifouling performance. A marked reduction in BSA adsorption was observed when the PHMG doping content reached 1 wt%, while further increasing the PHMG loading resulted in only minor additional decreases in protein adsorption.

### 3.8. Algae Resistance Analysis

**Figure 13:**
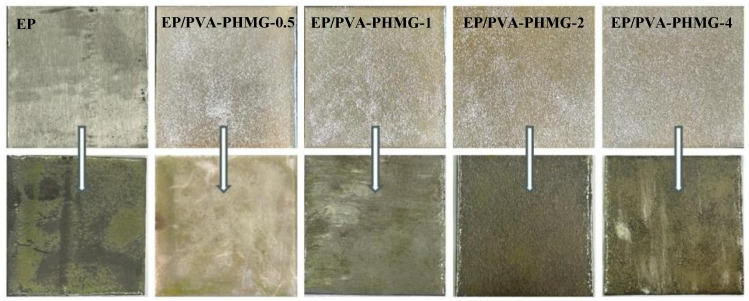
Algae resistance pictures of EP/PVA-PHMG-X.

As depicted in Figure 13, following 14 days of incubation, the neat epoxy resin coating displayed substantial algal colonization with obvious signs of adhered algae. In contrast, the EP/PVA-PHMG-1 coating exhibited only minor colonization by *Chlorella*. When the PHMG content exceeded 1 wt%, almost no algal adhesion was detected on the coating surfaces, indicating superior anti-algal activity. These results demonstrate that the introduction of 2 wt% PHMG solution significantly enhances the anti-algal performance of the coatings, thus endowing the composite coatings with excellent antifouling properties.

## 4. Conclusions

In this work, a novel antibacterial coating was successfully fabricated based on PVA cross-linked with PHMG. Benefiting from the introduction of PHMG, the resultant coating exhibited outstanding antibacterial activity, achieving more than 99.99% bactericidal efficiency against both *E. coli* and *S. aureus* at a PHMG content of 2 wt%. Moreover, the EP/PVA-PHMG-X coatings displayed excellent resistance to protein adsorption and effectively suppressed algal colonization. Given their combined superior antibacterial activity, protein-repellent ability, and anti-algal performance, the as-prepared EP/PVA-PHMG-X coatings hold great promise for practical applications in marine antifouling materials. The resultant coatings possess great potential for versatile applications on various substrates such as ship hulls, and can efficiently impede the attachment of marine microbes and the settlement of macro-fouling organisms. In this regard, the employment of such coatings contributes to reduced maintenance expenses and prolonged service life of marine facilities.

## Figures and Tables

**Table 1:** Proportioning of the sample components.

Samples	Composition (g)			
	10% PVA Solution (g)	20% PHMG Solution (g)	H_2_O (g)	GA (g)
PVA-PHMG-0.5	20	0.5	79.5	0.5
PVA-PHMG-1.0	20	1	79	1
PVA-PHMG-2.0	20	2	78	2
PVA-PHMG-4.0	20	4	76	4

**Table 2:** Leaching rate of PHMG (0.5 g samples immersed in 100 mL water).

Samples	Abs_192nm_	C_PHMG_ in Filtrate (mg/L)	Leaching Rate (%)
PVA-PHMG-0.5	0.2358	3.01	0.301
PVA-PHMG-1	0.4323	7.88	0.394
PVA-PHMG-2	0.4892	9.29	0.232
PVA-PHMG-4	0.5409	10.57	0.676

**Table 3:** TGA data of PVA and PVA-PHMG-X.

Samples	T_d5_ (°C)	T_dmax1_ (°C)	T_dmax2_ (°C)
PVA	319.1	333.3	452.6
PVA-PHMG-0.5	320.2	340.8	439.4
PVA-PHMG-1	322.5	349.7	433.8
PVA-PHMG-2	331.2	363	440.1
PVA-PHMG-4	340.1	366.1	430

**Table 4:** Water absorption capacity.

Samples	Weight Before Water Absorption (g)	Weight After Water Absorption (g)	Water Absorption (%)
EP/PVA-PHMG-0.5	12.67	12.95	280
EP/PVA-PHMG-1	13.01	13.27	260
EP/PVA-PHMG-2	11.79	12.03	240
EP/PVA-PHMG-4	12.90	13.07	170

**Table 5:** Antibacterial efficiency of micropowders against *E. coli* and *S. aureus* tested by the shaking flask method.

Samples	*E. coli* (%)	*S. aureus* (%)
PVA	0	0
PVA-PHMG-0.5	21.74	23.77
PVA-PHMG-1	80.17	84.32
PVA-PHMG-2	99.99	99.99
PVA-PHMG-4	99.99	99.99

**Table 6:** Antibacterial efficiency of coatings against *E. coli* and *S. aureus* tested by the film application method.

Samples	*E. coli* (%)	*S. aureus* (%)
EP	0	0
EP/PVA-PHMG-0.5	20.17	22.22
EP/PVA-PHMG-1	88.93	92.61
EP/PVA-PHMG-2	99.99	99.99
EP/PVA-PHMG-4	99.99	99.99

**Table 7:** Residual BSA absorbance, concentration, and adsorption amount on the coating surface.

Samples	Absorbance of 278 nm	Residual BSA Solution Concentration (g/L)	BSA Adsorption Capacity (μg/cm^2^)
EP	0.2658	0.4085	85.78
EP/PVA-PHMG-0.5	0.2731	0.4203	74.72
EP/PVA-PHMG-1	0.3096	0.4795	19.21
EP/PVA-PHMG-2	0.3165	0.4908	8.72
EP/PVA-PHMG-4	0.3187	0.4942	5.44

## Data Availability

The original contributions presented in this study are included in the article/Supplementary Material. Further inquiries can be directed to the corresponding author.

## Supplementary Materials

The following supporting information can be downloaded at: https://www.mdpi.com/article/10.3390/ma19071362/s1, Figure S1: (a) UV absorption spectra of aqueous solutions of PHMG at different concentrations and (b) the relationship between UV absorption intensity and concentration of PHMG; Figure S2: (a) Activation energy of the reaction between glutaraldehyde and propylamine and (b) reaction mechanism diagram; Figure S3: (a) Activation energy of the reaction between glutaraldehyde and guanidine hydrochloride and (b) reaction mechanism diagram; Figure S4: (a) UV absorption spectra of different concentrations of BSA and (b) the relationship between UV absorption intensity and concentration of BSA.

## References

[1] Wichterle O., Lím D. (1960). Hydrophilic gels for biological use. Nature.

[2] Tang T., Fei J., Wu S., He H., Ma M., Shi Y., Zhu Y., Chen S., Wang X. (2025). Biodegradable sodium lignosulfonate-based superabsorbent hydrogels for disposable hygiene products based on hyperbranched polyetherpolyol crosslinkers. Int. J. Biol. Macromol..

[3] Yegappan R., Selvaprithiviraj V., Amirthalingam S., Jayakumar R. (2018). Carrageenan based hydrogels for drug delivery, tissue engineering and wound healing. Carbohydr. Polym..

[4] Nguyen M.K., Lee D.S. (2010). Injectable Biodegradable Hydrogels. Macromol. Biosci..

[5] Niu Y., Wu J., Kang Y., Sun P., Xiao Z., Zhao D. (2023). Recent advances of magnetic chitosan hydrogel: Preparation, properties and applications. Int. J. Biol. Macromol..

[6] Zhao Y., Zhu Z.S., Guan J., Wu S.J. (2021). Processing, mechanical properties and bio-applications of silk fibroin-based high-strength hydrogels. Acta Biomater..

[7] Zhang Y., Wang J., Cui Z., Guo S., Wang Y., Li W., Zhou C., Run M., Qin J. (2024). Preparation of antibacterial hydrogel from poly(aspartic hydrazide) and quaternized N-[3-(dimethylamino) propyl] methylacrylamide copolymer with antioxidant and hemostatic effects for wound repairing. Colloids Surf. B Biointerfaces.

[8] Mo F., Jiang K., Zhao D., Wang Y., Song J., Tan W. (2021). DNA hydrogel-based gene editing and drug delivery systems. Adv. Drug Deliv. Rev..

[9] Xuan L., Hou Y., Liang L., Wu J., Fan K., Lian L., Qiu J., Miao Y., Ravanbakhsh H., Xu M. (2024). Microgels for cell delivery in tissue engineering and regenerative medicine. Nano-Micro Lett..

[10] Peppas N.A., Mongia N.K. (1997). Ultrapure poly(vinyl alcohol) hydrogels with mucoadhesive drug delivery characteristics. Eur. J. Pharm. Biopharm..

[11] Jiang Y., Yang Y., Zheng X., Yi Y., Chen X., Li Y., Sun D., Zhang L. (2020). Multifunctional load-bearing hybrid hydrogel with combined drug release and photothermal conversion functions. NPG Asia Mater..

[12] Martínez-Gómez F., Guerrero J., Matsuhiro B., Pavez J. (2017). In vitro release of metformin hydrochloride from sodium alginate/polyvinyl alcohol hydrogels. Carbohydr. Polym..

[13] Liu D., Cao Y., Jiang P., Wang Y., Lu Y., Ji Z., Wang X., Liu W. (2023). Tough, transparent, and slippery pva hydrogel led by syneresis. Small.

[14] Li N., Yu Q., Duan S., Du Y., Shi X., Li X., Jiao T., Qin Z., He X. (2024). Anti-swelling, high-strength, anisotropic conductive hydrogel with excellent biocompatibility for implantable electronic tendon. Adv. Funct. Mater..

[15] Liang X., Zhong H.-J., Ding H., Yu B., Ma X., Liu X., Chong C.-M., He J. (2024). Polyvinyl alcohol (pva)-based hydrogels: Recent progress in fabrication, properties, and multifunctional applications. Polymers.

[16] Lu G., Tian S., Li J., Xu Y., Liu S., Pu J. (2021). Fabrication of bio-based amphiphilic hydrogel coating with excellent antifouling and mechanical properties. Chem. Eng. J..

[17] Mao Q., Liu S., Xiong Y., Hu D., Huang L., Fang Z., Jiang H., Wang H., Li J., Mao S. (2023). Advanced marine antifouling hydrogels based on 7-amino-4-methylcoumarin fluorescence driven by rare-earth phosphorescence. ACS Appl. Mater. Interfaces.

[18] Lejars M., Margaillan A., Bressy C. (2012). Fouling release coatings: A nontoxic alternative to biocidal antifouling coatings. Chem. Rev..

[19] Fore M. (2020). Seeking nontoxic coatings to keep ship hulls clean. ACS Cent. Sci..

[20] Song B., Zhang E., Shi Y., Wang W., Zhu H., Gallagher S.J., Fischer S., Rigney J., Kim E., Cao Z. (2024). Zwitterionic hydrogel coating with antisediment properties for marine antifouling applications. ACS Appl. Mater. Interfaces.

[21] Xie Q., Pan J., Ma C., Zhang G. (2019). Dynamic surface antifouling: Mechanism and systems. Soft Matter.

[22] Wei Y., Li W., Liu H., Liu H. (2023). In situ preparation of spindle calcium carbonate-chitosan/poly (vinyl alcohol) anti-biofouling hydrogels inspired by shellfish. J. Ind. Eng. Chem..

[23] Erathodiyil N., Chan H.-M., Wu H., Ying J.Y. (2020). Zwitterionic polymers and hydrogels for antibiofouling applications in implantable devices. Mater. Today.

[24] He G., Liu W., Liu Y., Wei S., Yue Y., Dong L., Yu L. (2025). Antifouling hydrogel with different mechanisms: Antifouling mechanisms, materials, preparations and applications. Adv. Colloid Interface Sci..

[25] Chen H., Yang F., Chen Q., Zheng J. (2017). A novel design of multi-mechanoresponsive and mechanically strong hydrogels. Adv. Mater..

[26] Gutierrez-Alvarado K., Chacón-Cerdas R., Starbird-Perez R. (2022). Pectin microspheres: Synthesis methods, properties, and their multidisciplinary applications. Chemistry.

[27] Patel M.A., AbouGhaly M.H.H., Schryer-Praga J.V., Chadwick K. (2017). The effect of ionotropic gelation residence time on alginate cross-linking and properties. Carbohydr. Polym..

[28] Vallejo-Castillo V., Rodríguez-Stouvenel A., Martínez R., Bernal C. (2020). Development of alginate-pectin microcapsules by the extrusion for encapsulation and controlled release of polyphenols from papaya (*Carica papaya* L.). J. Food Biochem..

[29] Leong M.-H., Tan C.-P., Nyam K.-L. (2016). Effects of accelerated storage on the quality of kenaf seed oil in chitosan-coated high methoxyl pectin-alginate microcapsules. J. Food Sci..

[30] Jurišić Dukovski B., Mrak L., Winnicka K., Szekalska M., Juretić M., Filipović-Grčić J., Pepić I., Lovrić J., Hafner A. (2019). Spray-dried nanoparticle-loaded pectin microspheres for dexamethasone nasal delivery. Dry. Technol..

[31] Chacón-Cerdas R., Medaglia-Mata A., Flores-Mora D., Starbird-Pérez R. (2020). Synthesis of chitosan, pectin, and chitosan/pectin microspheres by two water-in-oil emulsion crosslinking methods. Chem. Pap..

[32] Pak C.W., Kosno M., Holehouse A.S., Padrick S.B., Mittal A., Ali R., Yunus A.A., Liu D.R., Pappu R.V., Rosen M.K. (2016). Sequence determinants of intracellular phase separation by complex coacervation of a disordered protein. Mol. Cell.

[33] Verkempinck S.H.E., Kyomugasho C., Salvia-Trujillo L., Denis S., Bourgeois M., Van Loey A.M., Hendrickx M.E., Grauwet T. (2018). Emulsion stabilizing properties of citrus pectin and its interactions with conventional emulsifiers in oil-in-water emulsions. Food Hydrocoll..

[34] Selim M.S., Shenashen M.A., El-Safty S.A., Higazy S.A., Selim M.M., Isago H., Elmarakbi A. (2017). Recent progress in marine foul-release polymeric nanocomposite coatings. Prog. Mater. Sci..

[35] Li W., Wu Q., Zhao X., Huang Z., Cao J., Li J., Liu S. (2014). Enhanced thermal and mechanical properties of pva composites formed with filamentous nanocellulose fibrils. Carbohydr. Polym..

[36] Han H., Zhu J., Wu D.-Q., Li F.-X., Wang X.-L., Yu J.-Y., Qin X.-H. (2019). Inherent guanidine nanogels with durable antibacterial and bacterially antiadhesive properties. Adv. Funct. Mater..

[37] Leong J., Yang C., Tan J., Tan B.Q., Hor S., Hedrick J.L., Yang Y.Y. (2020). Combination of guanidinium and quaternary ammonium polymers with distinctive antimicrobial mechanisms achieving a synergistic antimicrobial effect. Biomater. Sci..

