# Supplementary material for: Synthesis and Application of Polyvinyl Alcohol (PVA) Micropowders for Antifouling Coatings
Source: Materials (Basel). 2026 Mar 30;19(7):1362. doi: 10.3390/ma19071362 (PMC13074872; doi:10.3390/ma19071362)
Supplement: Supplementary file 1 [file materials-19-01362-s001.zip › materials-4176266-supplementary.pdf]

Supporting Information

S1. Ultraviolet Asorption Sectrum

A series of aqueous PHMG solutions with varying concentrations was prepared. The absorption spectra of these solutions were recorded over the wavelength range of 190–300 nm using UV–vis absorption spectroscopy, as displayed in Fig.S1 (a). A linear calibration curve was subsequently established, as shown in Fig.S1 (b).

$$Abs_{192nm}=0.11439+0.04035C_{PHMG}$$

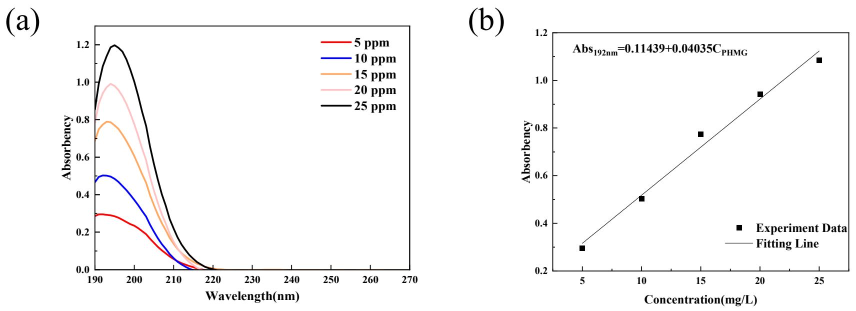

Fig.S1. (a) UV absorption spectra of aqueous solutions of PHMG at different concentrations and (b) the relationship between UV absorption intensity and concentration of PHMG.

As shown in Fig.S1, the absorption peak exhibits a slight shift with increasing PHMG concentration. This phenomenon can be attributed to the increased number of chromophores in the solution at higher PHMG loadings, which lowers the energy required for electronic transitions. Nevertheless, this small shift exerts negligible influence on the quantitative measurements and can thus be disregarded in the study.

S2. Gaussian Simulation

The activation energy and reaction mechanism were simulated using Gaussian software, with the corresponding results depicted in Fig.S2 and Fig.S3. According to the calculated activation energies, the reaction between glutaraldehyde and guanidine hydrochloride exhibits an activation energy of 31.14 kcal·mol^{-1}, which is considerably higher than that of the reaction between glutaraldehyde and propylamine. The relatively low activation energy of the glutaraldehyde–propylamine system indicates that this reaction can proceed readily at ambient temperature. In contrast, the markedly higher activation energy required for the glutaraldehyde–guanidine hydrochloride reaction implies a greater energy barrier for its initiation. These results demonstrate that the terminal amine groups act as the dominant reactive sites in PHMG, whereas the guanidine moieties are unlikely to participate directly in the cross-linking process. Accordingly, the data derived from the propylamine model are more representative of

the actual reaction between PHMG and glutaraldehyde.

The reaction proceeds via condensation between the amine group (–NH_2) and the aldehyde group (–CHO) of glutaraldehyde, initially forming an unstable α-hydroxyamine intermediate. This intermediate subsequently undergoes dehydration to yield a stable Schiff base. The remaining amine group is involved in the curing reaction with epoxy resin.

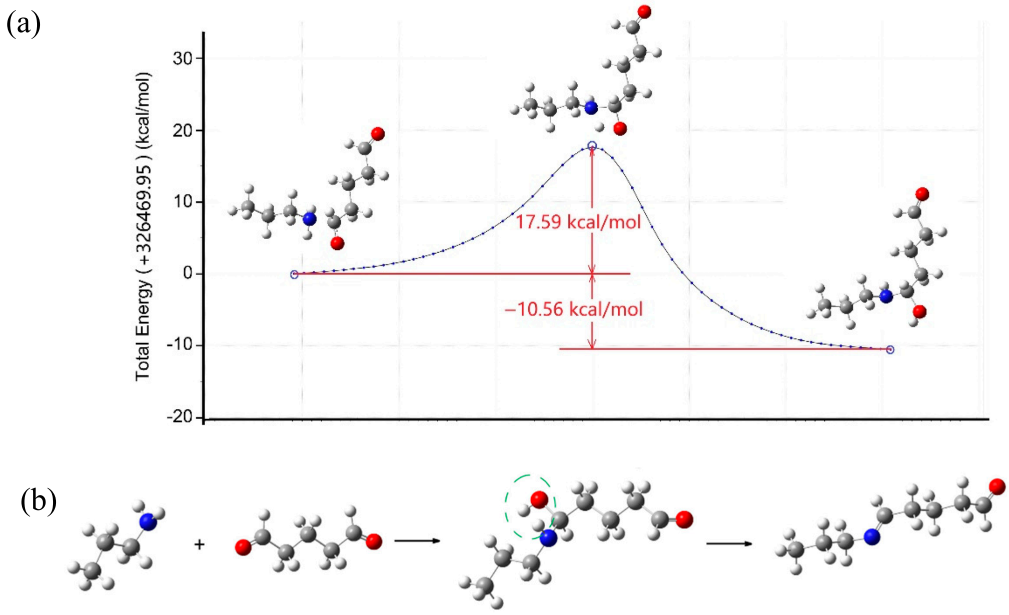

Fig.S2. (a) Activation energy of the reaction between glutaraldehyde and propylamine and (b) reaction mechanism diagram.

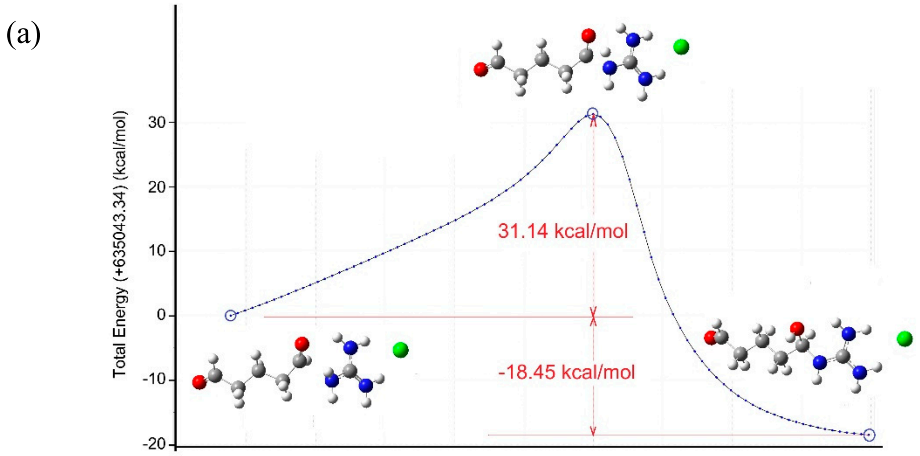

(b)

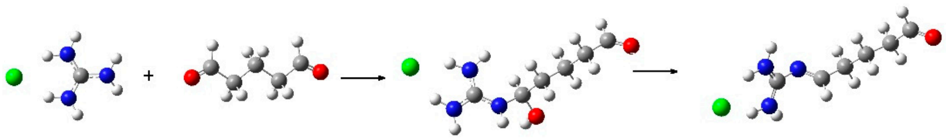

Fig.S3. (a) Activation energy of the reaction between glutaraldehyde and guanidine hydrochloride and (b) reaction mechanism diagram.

In the ball-and-stick model, white spheres represent hydrogen (H), gray spheres represent carbon (C), blue spheres represent nitrogen (N), red spheres represent oxygen (O), and green spheres represent chlorine (Cl).

S3. Anti-Protein Asorption Test

Based on the characteristic absorption of aromatic amino acids (e.g., tryptophan and tyrosine) in bovine serum albumin (BSA) at 278 nm, a series of BSA standard solutions with a concentration gradient of 5–25 ppm were prepared. The absorbance of each solution was measured at 278 nm, and a standard calibration curve (Fig.S4a) was established via least-squares linear fitting, yielding the quantitative relationship between absorbance and concentration (Fig.S4b).

(a) (b)

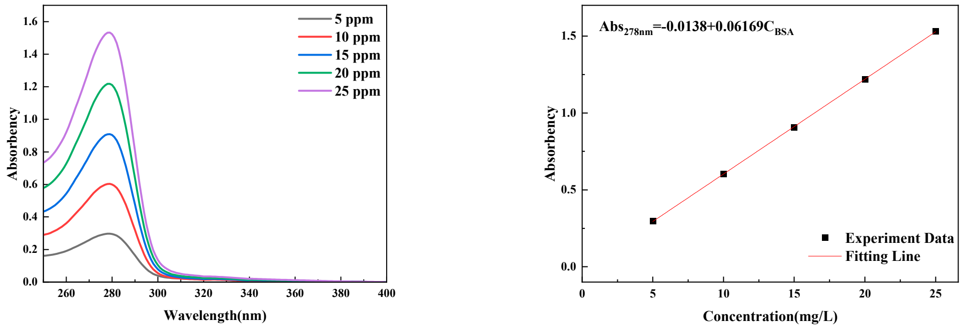

Fig.S4. (a) UV absorption spectra of different concentrations of BSA and (b) the relationship between UV absorption intensity and concentration of BSA.
